# How Gut Microbiota Are Shaped by Pattern Recognition Receptors in Colitis and Colorectal Cancer

**DOI:** 10.3390/cancers14153821

**Published:** 2022-08-06

**Authors:** Furong Qing, Tao Xie, Lu Xie, Tianfu Guo, Zhiping Liu

**Affiliations:** 1 Center for Immunology, Key Laboratory of Prevention and Treatment of Cardiovascular and Cerebrovascular Diseases, Ministry of Education, Gannan Medical University, Ganzhou 341000, China; 2 School of Graduate, Gannan Medical University, Ganzhou 341000, China; 3 Center for Scientific Research, Gannan Medical University, Ganzhou 341000, China; 4 School of Basic Medicine, Gannan Medical University, Ganzhou 341000, China

**Keywords:** pattern recognition receptor, gut microbiota, colitis, colorectal cancer, intestinal epithelium

## Abstract

**Simple Summary:**

The pathogenesis of intestinal inflammatory disorders such as colitis and colorectal cancer is complicated and dysregulation of gut microbiota is considered an important contributing factor. Inflammation is often initiated by the activation of pattern recognition receptors. However, the relationship between these innate immune receptors and gut microbiota is not fully understood. Here, we show that pattern recognition receptors not only recognize pathogens and initiate inflammatory signal transduction to induce immune responses, but also influence the composition of intestinal microorganisms, thus affecting the development of intestinal inflammation and cancer through various mechanisms. This suggests that the modification of innate immune receptors and relevant molecules could be therapeutic targets for the treatment of colitis and colorectal cancer by regulating gut microbiota.

**Abstract:**

Disorders of gut microbiota have been closely linked to the occurrence of various intestinal diseases including colitis and colorectal cancer (CRC). Specifically, the production of beneficial bacteria and intestinal metabolites may slow the development of some intestinal diseases. Recently, it has been proposed that pattern recognition receptors (PRRs) not only recognize pathogens and initiate inflammatory signal transduction to induce immune responses but also influence the composition of intestinal microorganisms. However, the mechanisms through which PRRs regulate gut microbiota in the setting of colitis and CRC have rarely been systematically reviewed. Therefore, in this paper, we summarize recent advances in our understanding of how PRRs shape gut microbiota and how this influences the development of colitis and CRC.

## 1. Introduction

Inflammatory bowel diseases (IBD) are chronic, etiologically unclear, and recurrent intestinal inflammatory diseases resulting from disruption of intestinal homeostasis by abnormal immune responses to microbial and environmental factors in genetically susceptible individuals, mainly including Crohn’s disease (CD) and ulcerative colitis (UC) [1]. The pathogenesis of IBD involves dysbiosis of the gut microbiota, genetic susceptibility, and immune abnormalities [2]. IBD is a well-known risk factor for colorectal cancer (CRC) [3].

According to the International Agency for Research on Cancer (IARC), in 2020, CRC ranked third in the number of new cancer cases (10.0%) and second in the number of deaths (9.4%) [4]. Although overall CRC-related morbidity and mortality rates have continued to decline in recent years, the number of young adult CRC cases is on the rise, and the IARC expects the prevalence of CRC to increase significantly by 2070 [5]. CRC is undoubtedly one of the most common malignant tumors of the digestive tract.

The pathogenesis of CRC is widely believed to be related to various factors, including genes, environment, and chronic inflammation. Gut microbiota composition and metabolites play important roles in the pathogenesis of CRC. Aberrant immune responses to gut microbiota can also lead to colitis and even CRC [6].

Pattern recognition receptors (PRRs) are a class of non-clonally distributed receptors that are expressed mainly on innate immune cells. PRRs such as the Toll-like receptors (TLRs), Nod-like receptors (NLRs), Aim2-like receptors (ALRs), RIG-I-like receptors (RLRs), and C-type lectin-like receptors (CLRs) primarily recognize pathogen-associated molecular patterns (PAMPs) or danger-associated molecular patterns (DAMPs); their activation activates a cascade of downstream signals ultimately resulting in the production of proinflammatory cytokines.

Given the important role of PRRs in innate immunity, PRRs may directly or indirectly influence the gut microbiota; PRR dysfunction is associated with intestinal inflammation and tumorigenesis. However, the effects and mechanisms of PRRs on gut microbiota remain controversial.

Given the complexity and controversy associated with PRRs in this context, this review summarizes recent research advances on the role of how PRRs shape gut microbiota and how this relates to the development of colitis and CRC. Since the relationship between TLRs and gut microbiota in the setting of colitis and CRC has been reviewed [7,8], we will focus on the interaction between other PRRs and gut microbiota during the development of intestinal inflammation and tumorigenesis.

## 2. Nod-like Receptors

The NLR family includes three main sub-families: the Nods (nucleotide-binding oligomerization domain containing 1 (Nod1), nucleotide-binding oligomerization domain containing 2 (Nod2) [9], nucleotide-binding oligomerization domain containing 3(Nod3)/nucleotide-oligomerization domain-like receptor subfamily C3 (Nlrc3) [10], nucleotide-binding oligomerization domain containing 4 (Nod4), nucleotide-binding oligomerization domain containing 5 (Nod5)/nucleotide-binding domain and leucine-rich repeat-containing protein X1 (Nlrx1) [11], and Class II major histocompatibility complex transactivator (CIITA), the Nlrps (Nlrp1–14, also known as NACHT-LRR-PYD-containing proteins (Nalps) [11]), and the Ipaf subfamily (nucleotide-oligomerization domain-like receptor subfamily C4 (Nlrc4) [12] and neuronal apoptosis inhibitory protein (Naip) [13]). The inflammasome generally consists of NLRs, adaptor apoptosis-associated speck-like protein(ASC), and caspase-1, which regulate the maturation and secretion of the proinflammatory cytokines IL-1β and IL-18, and initiate inflammatory processes [14].

## 3. Nod1 Receptor

Nod1 can enhance the epithelial barrier function, promote intestinal homeostasis, and resist the invasion of pathogens that can cause colitis by regulating antimicrobial peptides (AMPs), proinflammatory cytokines, autophagy, and acquired immunity [15]. *Salmonella*, for example, activates Nod1 in the gut through peptidoglycan (PGN) motifs which are the main component of its bacterial cell wall; the intestinal epithelium thus acts as a barrier against pathogen invasion, thereby limiting the severity of colitis or CRC. However, studies testing genetic coding polymorphisms of Nod1 have failed to identify an increased susceptibility to colitis [16,17]; as such, these conflicting views need further investigation.

In a mouse CRC model induced by the mutagen Azomethane (AOM) or the inflammatory agent sodium dextran sulfate (DSS), *Nod1−/−* mice show more severe colitis or tumor formation than wild-type (WT) mice. This is related to the increased apoptotic responses and damage of intestinal epithelial cells, the lower integrity of the intestinal epithelial barrier, and the altered composition of the gut microbiota. Specifically, *Nod1−/−* mice show poor neutrophil mobilization at the site of infection resulting in increased bacterial translocation in the intestinal tract, and reduced secretion of IL-6 and IFN-β, thereby affecting intestinal epithelial barrier function [18,19]. By using cohousing and fecal transplantation models, it has been further shown that 16S rRNA sequenced gut microbiota populations of *Nod1−/−* mice were different from controls [15,20]. Mice with Nod1 gene defects have different relative numbers of bacteria, including *Clostridium*, *Bacteroids*, and *Enterobacteriaceae* [21]. In addition, antibiotic treatment of Nod1 gene-deficient mice significantly inhibits the formation of intestinal tumors, suggesting that gut microbiota play an important role in the development of intestinal tumors [22], although the specific pathogenic microbial species have not been elucidated.

## 4. Nod2 Receptor

Nod2 is the first identified CD susceptibility gene, although it does not alter the susceptibility to UC; this phenomenon has been demonstrated in German, Australian, and UK populations [23]. Nod2 is mostly expressed by monocytes, macrophages, dendritic cells, and intestinal Paneth epithelial cells. It is a cytoplasmic receptor that recognizes the presence of cell wall components of Gram-positive and negative bacteria, dipeptide (MDP) [24,25]. Upon recognition of bacterial MDP by Nod2, a common protein kinase, RIP2, is recruited, which in turn activates NF-κB and mitogen-activated protein kinase (MAPK) signaling pathways, leading to the expression of proteins such as AMPs, cytokines, and chemokines that contribute to the strengthening of the epithelial barrier [26]. Nod2 is involved in maintaining the balance of intestinal barrier integrity, immune homeostasis, autophagy, and gut microbiota composition thus limiting bacterial displacement [27].

Nod2 deficiency increases host susceptibility to colitis; this is associated with altered gut microbiota. Dysbiosis is associated with reduced intestinal epithelial barrier integrity and increased permeability [20]. It has been shown that *Nod2−/−* mice have a significantly reduced proportion of *Lactobacillus* and *Clostridium perfringens* in the gut and *Bacteroidetes* belonging to the *Porphyromonadaceae* family are lower in the colon of *Nod2−/−* mice compared to WT mice, while the proportion of *Firmicutes*, *Clostridium perfringens IV* and *Aspergillus* from the genus *Lachnospiraceae* are higher [27]. Analysis of germ-free (GF) mice colonized with fecal homogenate from SPF *Nod2−/−* mice shows a greater preponderance for *Bacteroides* and less for *Butyrivibrio* and *Lachnobacterium* than after fecal homogenates from SPF WT mice. WT mice co-housed with *Nod2−/−* mice show greater weight loss, more severe bloody diarrhea, higher histological scores stemming from more severe colonic crypt destruction and epithelial surface ulceration, and higher mortality and morbidity after DSS administration, compared to WT mice housed alone. However, antibiotic treatment eliminates these effects [28]. It appears that recolonization of WT mice with fecal microbiota from *Nod2−/−* mice raise the risk of disease. Moreover, *Nod2−/−* mice exhibit more severe colitis and an increased abundance of intestinal *Aspergillus*, *E. coli*/*Shigella*, and *Enterococcus* spp. compared to co-caged WT mice [27,29]. Similar results were obtained via embryo transfer experiments [25].

Based on the above effects on the gut microbiota of Nod2 knockout, the following four mechanisms may be involved.

**Figure 1:**
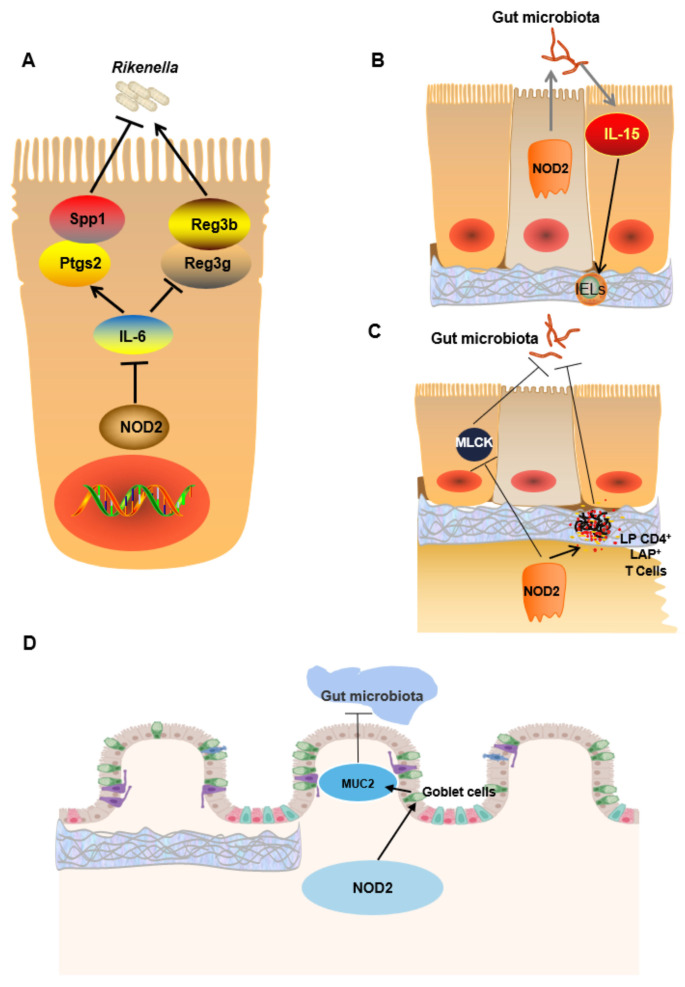
Nod2 mediated regulation of gut microbiota. (**A**) Nod2 in epithelial cells inhibits the expression of IL-6 and subsequently Ptgs2 and Spp1, and increases the expression of Reg3β and Reg3γ, thus increasing the abundance of *Rikenella*. (**B**) Nod2 maintains IELs via recognition of gut microbiota. The loss of IELs in *Nod2−/−* mice is caused by the impaired expression of IL-15. (**C**) Nod2 can inhibit the overexpression of MLCK, avoiding the opening of tight junctions of epithelial cells, decreasing permeability of intestinal epithelial mucosa and bacterial translocation. Nod2 can also affect the number of LP CD4^+^LAP^+^ T cells, and regulate gut microbiota and improve colitis. (**D**) Nod2 can promote the secretion of Muc2 by intestinal goblet cells and enhances intestinal barrier function, thereby limiting bacterial displacement.

First, Nod2 mediates the expression of downstream molecules and the release of AMPs through the IL-6 signaling pathway, thereby affecting the gut microbiota. Loss of Nod2 or RIP2 results in a proinflammatory microenvironment that enhances epithelial dysplasia following chemically induced injury. IL-6 mediated signals are upregulated in *Nod2−/−* mice, which leads to increased expression of prostaglandin peroxide synthase 2 (Ptgs2), secretory phosphoprotein 1 (Spp1), and trigonelline factor 3 recombinant protein (TFF3) and the immunoglobulin binding protein Fc fragment (Fc-γBp), and decreased expression of Reg3β, Reg3γ, and phospholipase A2 [25,30]. Anti-IL-6 receptor treatment of *Nod2−/−* mice is associated with a higher proportion of *Rikenella*, and antibiotic treatment significantly reduces genotype-driven morbidity and mortality (Figure 1A). Thus, the composition of *Nod2−/−* mouse mucosa-associated bacterial communities can be reversed by antibiotics or anti–IL-6 receptor treatment [30].

Second, Nod2 regulates the number of intestinal intraepithelial lymphoid cells (IELs) and affects the secretion of intestinal AMPs and other antimicrobial substances, which in turn affects the gut microbiota. Nod2 expression in the intestine is dependent on the presence of gut microbiota, and gut microbiota are required for the maintenance of IELs. Nod2 deficiency significantly decreases IEL numbers. IELs can be significantly recovered via supplementation of the Nod2 agonist MDP, but not via the supplementation of the Nod1 agonist iEDAP. MDP increases the number of IELs by increasing the expression of the pro-proliferative cytokine IL-15. IELs can secrete some antimicrobial compounds and thus have a killing effect on some gut microbiota. Consequently, decreased numbers of IELs in *Nod2−/−* mice might induce the alteration of gut microbiota (Figure 1B) [31].

Third, Nod2 affects the gut microbiota by mediating the number of LP CD4^+^LAP^+^ T cells and myosin light chain kinase (MLCK) activity. *Nod2−/−* mice have increased tumor necrosis factor alpha (TNF-α) and interferon-gamma (IFN-γ) which induces overexpression of MLCK. MLCK phosphorylates ser19 and thr18 of the myosin light chain, changing its spatial conformation and thus promoting the contraction of actin and myosin filaments, opening up tight junctions in epithelial cells, increasing intestinal epithelial mucosal permeability, and increasing bacterial translocation thereby altering the number of LP CD4^+^LAP^+^ T cells [32]. This suggests that Nod2 can inhibit the overexpression of MLCK, avoid the opening of tight junctions of epithelial cells, and decrease the permeability of intestinal epithelial mucosa and bacterial translocation (Figure 1C). LP CD4^+^LAP^+^ T cells are regulatory T cells that ameliorate colitis by exerting corresponding immune effects. It has been suggested that LP CD4^+^LAP^+^ T cells are involved in modulating the gut microbiota and reducing the severity of ethanol- and TNBS-induced colitis [33]. However, no significant differences were found in the number of LP CD4^+^LAP^+^ T cells in DSS-induced colitis. This suggests that other mechanisms may be important in regulating gut microbiota.

Fourth, Nod2 can affect the gut microbiota by modulating the number of goblet cells in the intestinal epithelium. Nod2 knockout reduces goblet cells in colonic mucosa and Muc2 secretion, resulting in an impaired intestinal epithelial barrier, greater bacterial translocation, imbalance of bacterial communities, and increasing the host’s susceptibility to colitis and CRC [34]. This indicates that Nod2 can promote the secretion of Muc2 by intestinal goblet cells and enhances intestinal barrier function, thereby limiting bacterial displacement (Figure 1D).

Although some early studies showed that *Nod2−/−* mice are more susceptible to DSS-induced colitis and that this bears no relationship with disordered gut microbiota, there has been increasing data suggesting a relationship between *Nod2−/−* mice and gut microbiota. Overall, Nod2 can affect the gut microbiota by mediating the IL-6 pathway, the number of IELs, and goblet cells, MLCK activity, and the number of LP CD4^+^LAP^+^ T cells, but the causal links remain to be definitively identified.

## 5. Nlrp3 Receptor

Nlrp3, a typical member of the NLRP-subfamily, contains PYD, NACHT, and LRR domains that recruit ASC and caspase-1 precursors to form the inflammasome, which leads to the maturation and secretion of IL-1β and IL-18. IL-1β can maintain the bacterial balance while IL-18 can promote the integrity of the intestinal epithelial barrier [21,35]. Studies have shown that compared to WT mice, *Nlrp3−/−* mice are more susceptible to DSS-induced colitis and AOM/DSS-induced CRC, and that these mice show a reduced expression of β-defensin, IL-1β, IL-10, TGF-β, and altered microbiota (even as far as the development of bacteremia) [36]; for example, *Rikenellaceae, Enterobacteriaceae, Mycobacterium, Clostridium,* and *Lactobacillus* are increased while *Bacteroidaceae*, *Verrucomicrobia,* and *Akkermansia* are decreased (Table 1) in *Nlrp3−/−* mice [37,38]. Treatment with antibiotics reduces the severity of colitis symptoms in *Nlrp3−/−* mice compared to *Nlrp3−/−* mice that are not treated with antibiotics [36]. *Staphylococcus aureus* has been shown to suppress colitis and related CRC by activating Nlrp3 [39], and *Bacillus fragilis* inhibits the secretion of IL-1β and IL-18 regulated by Nlrp3 [40]. A mutation at a site in the functional coding region of Nlrp3 (*Nlrp3*^R258W^) can reshape the composition of gut microbiota (such as decreasing *Actinobacteria, Verrucomicrobia,* and *Akkermansia* while increasing *Lactobacillus*) by enhancing the production of IL-1β and the secretion of AMPs. Furthermore, this Nlrp3 mutation increases the number of locally potent anti-inflammatory Treg cells that maintain intestinal homeostasis and protect the host against experimental colitis and CRC [41].

In general, both overexpression and deletion of Nlrp3 lead to the production of AMPs and changes in gut microbiota, which may be critical for the development of colitis and CRC. However, the relationship between Nlrp3 and the gut microbiota still needs further validation. Variables such as the feeding environment of the control mice, the loading and composition of the pre-existing microbiota, and the genetic background of the mice need to be carefully tested.

## 6. Nlrp6 Receptor

Nlrp6 is an NLR that is expressed in intestinal epithelial cells; it interacts with ASC and caspase-1/caspase-11 to form the inflammasome. Like Nlrp3, the Nlrp6 inflammasome also senses microbial ligands or cellular stress signals via its nucleotide-binding domain (NBD) and activates caspase-1-mediated maturation of IL-1β and IL-18. The protective mechanism mediated by Nlrp6 may be related to the production of IL-18. The reduced secretion of IL-18 after Nlrp6 knockout weakens the ability of intestinal epithelial cells to recover after DSS-induced injury and affects the expression of the AMPs gene in the intestinal tract, thereby imparting greater disease susceptibility in these animals than in controls [45,46,59]. Previous studies have shown that *Nlrp6−/−* mice show gut microbiota dysbiosis compared to non-littermate WT mice [49]. A further study showed that the Nlrp6-IL-18-AMP axis is involved in the regulation of the microbiota composition and that its absence drives dysbiosis [60], suggesting that ecological dysregulation caused by Nlrp6 deficiency is responsible for increased host susceptibility to colonic inflammation. For example, in *Nlrp6−/−* mice, *Akkermansia muciniphila* were shown to colonize the intestinal tract, further contributing to the development of colitis [46]. In addition, intestinal dysbiosis of *Prevotella*, *TM7*, *Firmicutes,* and *Bacteroidetes* was found in *Nlrp6−/−* mice [47,48,49].

Based on some of the previous studies, a research group conducted a controlled trial and found that biological disorders caused by Nlrp6 and their corresponding changes do not play a major role in colitis pathogenesis [61]. It was also pointed out that there was no significant difference between WT and *Nlrp6−/−* mice in terms of gut microbiota, but that a sex-dependent microbial community structure appeared. For example, intestinal *Bacilli*, *Lactobacilli,* and *Proteobacteria* were more common in female mice, and *Premicutes*, *Firmicutes*, *Actinobacteria,* and *Tenericutes* were more common in the intestinal tracts of male mice [47].

In conclusion, Nlrp6 may affect the development of colitis or CRC by altering the composition of intestinal bacteria and the function of intestinal epithelial cells, but the same results are not obtained in the same nest control experiment. This finding suggests that the role of Nlrp6 in shaping intestinal microbial ecology and altering host susceptibility to DSS-induced colitis needs further validation.

## 7. Nlrp12 Receptor

Nlrp12 has been shown to regulate the production of inflammatory cytokines, such as suppressing TNF and IL-6 signal transduction, and both typical and atypical NF-κB signaling exert anti-inflammatory effects [62]. Nlrp12 is also involved in maintaining gut microbiota homeostasis, promoting the growth of beneficial bacteria, and playing a protective role in colitis and related tumors, which may be related to its anti-inflammatory activity [51]. Studies have shown that *Nlrp12−/−* mice are more susceptible to DSS-induced colitis and have a lower diversity of intestinal microbial communities than WT mice, but the performance of germ-free (GF)-*Nlrp12−/−* mice is similar to that of GF-WT mice. The expression of Nlrp12 in the bone marrow determines the diversity of gut microbiota. The abundance of *Bacteroidales*, *Clostridiales,* and *Lachnospiraceae* is decreased in *Nlrp12−/−* mice, while the abundance of *Erysipelotrichaceae* is increased. However, the suppression of pro-inflammatory cytokines, (e.g., via anti-TNF and anti-IL-6R antibody treatment) restores *Nlrp12−/−* induced changes in gut microbiota and alleviates the severity of colitis [51]. In addition, Nlrp12 is found to be downregulated in colitis patients [51].

## 8. Aim2 Receptor

Aim2 is a cytoplasmic innate immune receptor, which binds to dsDNA, enforces ASC, and activates caspase-1 to form the inflammasome. The Aim2 inflammasome mediates the secretion of IL-1β and IL-18 and contributes to host defenses against some viral and bacterial infections [53,63]. Meanwhile, Aim2-mediated IL-18 release promotes the secretion of IL-22 binding protein and AMPs, thus regulating intestinal homeostasis [52]. A study showed that compared with WT mice, *Aim2−/−* mice are more susceptible to DSS-induced colitis and have more severe symptoms such as weight loss; antibiotics significantly attenuate this phenotype [64]. Studies have shown that an intestinal microbiome imbalance enhances the susceptibility of *Aim2−/−* mice to CRC. The 16S rRNA gene sequencing of the gut microbiota showed that the abundance of intestinal *Akkermansia muciniphila* and *Anaeroplasma* is increased, while *Anaerostipes*, *Bifidobacterium*, *Flexispira*, *Prevotella,* and *Paraprevotella* are decreased in *Aim2−/−* mice in comparison with controls [53,65].

**Figure 2:**
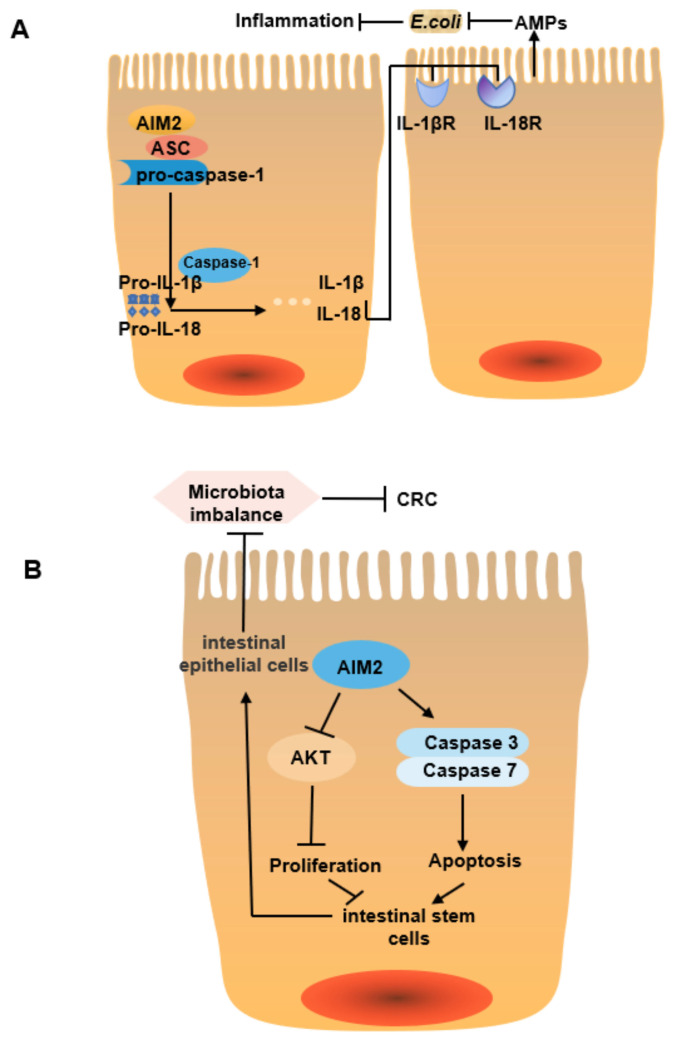
The pathway for Aim2 mediated regulation of microbiota. (**A**) Aim2 in intestinal epithelial cells (IECs) is activated by microbiota dsDNA in the cytoplasm and assembles the inflammasome by recruiting ASC and pro-caspase-1. The Aim2 inflammasome activates caspase-1 and induces the maturation of IL-1β and IL-18, which bind to their respective receptors IL-1βR and IL-18R on epithelial and immune cells, thereby increasing the production of AMPs to inhibit *E. coli* and reducing host susceptibility to colitis. (**B**) Aim2 can inhibit AKT and activate caspase3/7 signaling pathway to regulate cell proliferation and apoptosis, thus preventing intestinal stem cells from overproliferating and thus differentiating to form mature intestinal epithelial cells, thereby inhibiting gut microbiota translocation disorder and tumorigenesis.

Based on the aforementioned effects of an Aim2 knockout, these mechanisms may include the following two points. First, Aim2 can induce the production of IL-18 and IL-1β, thereby promoting the expression of AMPs, thus regulating gut microbiota and inflammation. It has been found that the pathogenic gut microbiota load is higher in *AIM2−/−* mice compared to WT mice, mainly due to an increased abundance of *E. coli*; stimulation of intestinal epithelial cells with IL-18 and IL-1β in vivo and in vitro promotes, the expression of AMPs such as Reg3β, Reg3γ, S100A9 and Lcn2 and effectively kills *E. coli*, thereby regulating inflammation [64] (Figure 2A). Second, Aim2 can activate AKT and caspase3/7 signaling pathways to regulate cell proliferation and apoptosis, thus affecting the permeability of intestinal epithelial cells and the expression of AMPs, which in turn regulates gut microbiota and tumorigenesis. It has been found that after AOM/DSS treatment, *Aim2−/−* mice have more BrdU^+^ and Ki67^+^ proliferative cells in each colonic crypt, increased activation of AKT signaling, and decreased activation of caspase3/7 signaling, resulting in increased transcription of proliferation-related genes and decreased apoptosis of cells (especially intestinal stem cells); such a state of excessive intestinal stem cells proliferation can predispose to CRC. In addition, the formation of immature intestinal epithelial cells by differentiation impairs intestinal epithelial barrier function, decreases the expression of AMPs, and increases the translocation of gut microbiota, resulting in a more disease-susceptible state [53]. This suggests that Aim2 can inhibit AKT and activate the caspase3/7 signaling pathway to regulate cell proliferation and apoptosis, thus preventing intestinal stem cells from overproliferation and also altering gut microbiota composition, therefore inhibiting tumorigenesis (Figure 2B).

In conclusion, Aim2 can influence intestinal inflammation and cancer development by regulating the gut microbiota in an inflammasome-dependent or independent manner. The relationships between these two pathways and whether they contradict each other remain to be further investigated.

## 9. RIG-I-like Receptor

Retinoic acid-inducible gene I (RIG-I) is a cytoplasmic receptor that specifically recognizes dsRNA and recruits MAVS, triggering downstream activation of Irf3/7 and NF-κB signaling pathways, thus inducing the production of IFN-Ⅰ and related proinflammatory cytokines [66,67]. Studies have shown that Rig-I expression is decreased in CRC patients and AOM/DSS-treated mice, and that *Rig-I−/−* mice are more susceptible to AOM/DSS-induced CRC [54]. Interestingly, the species richness and diversity of gut microbiota in *Rig-I−/−* mice is higher than in WT mice, possibly due to defects in IL-6-STAT3-dependent Reg3γ expression and IgA antibody production in the intestinal mucosa of *Rig-I−/−* mice [54]. However, specific bacterial species that migrate due to Rig-I defects have not been identified. After AOM/DSS treatment, *Rig-I−/−* mice given an antibiotic cocktail were found to be more susceptible to colitis, suggesting that intestinal microbiome imbalance is not the main cause of the high susceptibility and that Rig-I mediated signaling may play a major role in determining the host susceptibility to CRC [54]. The molecular mechanisms might involve an association with suppression of immune activity in Rig-I-deficient mice at early stages of DSS treatment and enhancement of tissue injury at later stages of exposure.

## 10. Cyclic GMP-AMP Synthase (cGAS) and Stimulator of Interferon Genes (Sting) Receptor

The cGAS/Sting signal axis includes cGAS and Sting. cGAS and Sting activation leads to the recruitment of TBK1; IRF3 and NF-κB are then phosphorylated, thus inducing the expression of IFN-I and other pro-inflammatory cytokines to regulate host defenses against microbial infection [55,68,69]. It was shown that compared to WT mice, *Sting−/−* mice are more susceptible to AOM/DSS induced CRC, have less intestinal mucus secretion and IgA production, reduced goblet cells, and reduced secretion of IL-1β and IL-10. In addition, *Sting−/−* mice display an altered gut microbiota composition, demonstrated by less *Bifidobacterium* and *Actinomycetes*, and higher *Desulphurvibrio* and *Proteus* [55,56]. Therefore, *Sting* plays an important role in maintaining intestinal barrier function and homeostasis. In addition, antibiotic treatment of *Sting−/−* mice reduces the severity of colitis, suggesting that gut microbiota play an important role in increasing the severity of colitis in *Sting−/−* mice [56].

## 11. C-Type Lectin-like Receptors

CLRs are a family of cell membrane innate immune receptors that bind to carbohydrates and mediate the recognition of fungal pathogens and subsequent initiation of antifungal immune responses [70]. CLRs include Dectin-1, Dectin-2, Dectin-3, and Mincle.

Studies have shown that Dectin-1 and Dectin-3 play an important role in the prevention of colitis. A loss of Dectin-1 signaling reduces the production of AMPs including S100A8 and S100A9, which increase intestinal *Lactobacillus murinus* and host susceptibility to colitis-associated CRC [57]. Dectin-3 deficiency impairs the phagocytic and bactericidal ability of macrophages, NF-κB activation, the production of proinflammatory cytokines (such as IL-6, TNF-α, and Th17), and the ability of intestinal epithelial tissue to repair. In addition, Dectin-3 deficiency promotes the number of intestinal *Candida tropicalis* and aggravates the severity of DSS-induced colitis [58].

## 12. Conclusions

Microbial and host factors along with environmental or other lifestyle factors all contribute to the development of cancer [71]. Among them, gut microbiota have been shown to play critical roles in CRC development.

PRRs are known to initiate immune responses in response to the recognition of ligands from gut microbiota. PRRs are associated with the development of colitis and CRC, thus suggesting that they are novel therapeutic targets.

PRRs can modulate the profiles of gut microbiota via different regulatory mechanisms. Some PRRs (such as Nlrp3, Nlrp6, and Aim2) can form inflammasomes and modify the components of gut microbiota mainly by regulating the production of IL-1β or IL-18 [72], while others (such as Nod1, Nod2, and Nlrp12) regulate it through the IL-6 pathway [51]. Moreover, RIG-I can modify gut microbiota composition by regulating the functions of intestinal epithelial cells [54], and Nod2 has the function of regulating specific T cell populations (such as LP CD4^+^ LAP^+^ T cells) [33].

The inflammasome assembled by some PRRs, ASC, and caspase-1 can mediate the maturation of IL-1β and IL-18. They can activate immune cells, and induce the production of other cytokines, chemokines, and growth factors, which promote proliferation and repair of intestinal epithelial cells to maintain mucosal integrity, thus effectively alleviating the development of colitis and CRC [73]. In addition, some PRRs can inhibit the occurrence and development of CRC by suppressing the activity of intestinal stem cells [74]. However, the over-activation of PRRs may cause excessive and recurrent inflammation and enhance the development of CRC.

Encouragingly, the regulation of PRRs may be utilized for the treatment of CRC. For example, activation of RIG-I promotes the integrity of the intestinal epithelium treated with irradiation and reduces the severity of graft versus host disease [67]. Some PRRs also respond to radiotherapy DNA damage and mediate protective immune responses. For example, AIM2 responds to ionizing radiation-induced DNA breaks in the nucleus and drives the proliferation of IECs, whereas STING senses dying tumor DNA after radiation to exert a protective effect during radiotherapy [74].

In addition, modification of gut microbiota by probiotics can facilitate the host to maintain the balance between gut microbiota and immune system, and resist the infection of pathogenic bacteria. Firstly, probiotics can enhance intestinal barrier function. For example, *Lactobacillus* regulates the expression of *Cadherin* and *β-catenin* and enhances intestinal barrier function [75]. Probiotics are also able to repair the epithelial barrier after injury [76,77]. Secondly, probiotics have strong adhesion to the intestinal mucosa and can prevent the attachment of pathogenic bacteria. The surface proteins of probiotics can compete with pathogenic bacteria, limiting their adhesion [78]. Thirdly, probiotics can produce beneficial substances such as organic acids, short-chain fatty acids, and AMPs, which inhibit pathogenic bacteria proliferation, thus enhancing the integrity of the intestinal epithelium [79,80,81]. Lastly, probiotics participate in the regulation of the immune system and thus promote the host’s resistance to pathogenic bacteria infection [82]. Probiotics can also regulate the expression of PRRs. For instance, probiotics can downregulate the transcription of *Clec7a*, which in turn affects the expression of *Dectin-1* by macrophages, thus enhancing the phagocytosis capability of macrophages [83].

However, there are limitations in the existing studies of the interactions between PRRs and gut microbiota. Inadequate evidence exists to suggest that changes in gut microbiota profiles will lead to significant phenotypic alterations. Meanwhile, there are inconsistencies in findings surrounding the relationship between PRRs, such as Nlrp6, and altered gut microbiota [61]. Such discrepancies may stem from non-genetic confounders such as maternal inheritance and long-term separate housing. Therefore, littermate-controlled experimental designs are critical to properly elucidate host–microbe interactions.

Numerous studies have suggested that PRRs could regulate gut microbiota composition, thus affecting the development of intestinal inflammation and cancer. This also indicates that modification of PRRs and relevant molecules could be therapeutic targets for the treatment of colitis and CRC by regulating gut microbiota. Indeed, most of these studies are performed in mice and may not be applicable to humans. The utilization of human fecal microbiota and humanized mice will be beneficial for studying the interplay between PRRs and gut microbiota, which will be more suitable for clinical application.

In conclusion, the mechanisms underlying how PRRs regulate gut microbiota profiles require careful exploration and validation. More efforts are still needed to properly characterize the relationship between PRRs and gut microbiota and clarify the mechanisms through which the host can be protected from the development of colitis and CRC.

## Figures and Tables

**Table 1:** Alteration of gut microbiota by PRRs.

PRRs	Possible Mechanisms	Microbiota Changes
Nod1	Nod1 is a cytoplasmic receptor that can recognize iE-DAP, activate NF-κB and MAPK signals, regulate AMPs, proinflammatory cytokines, autophagy, and acquired immunity to enhance epithelial barrier function, promote intestinal homeostasis, and resist the invasion of pathogens that may cause colitis. Nod1 knockout enhances the damage and apoptotic responses of intestinal epithelial cells, enhances intestinal epithelial permeability, reduces epithelial barrier, and increases bacterial abundance [42], thereby increasing susceptibility to colitis. However, antibiotic treatment of Nod1 knockout mice significantly inhibits the formation of intestinal tumors, suggesting that gut microbiota plays an important role in the development of colorectal associated tumors; the specific microbial species have not been elucidated [19,22].	The number of bacteria has increased, but the specific species are not characterized.
Nod2	Nod2 is a cytoplasmic receptor that can recognize MDP and recruit RIP2, thereby activating NF-κB and MAPK signaling pathways. Nod2 also plays an important role by promoting the production of antibacterial compounds (including defensin) in Paneth cells. After Nod2 knockout, the production of AMPs decreases, and the tumor necrosis factor α and interferon γ increase, resulting in the overexpression of MLCK. MLCK phosphorylates Ser19 and Thr18 of the myosin light chain, changing its spatial conformation, promoting the contraction of actin and myosin filaments, and opening epithelial cell contacts, thus increasing the permeability of the intestinal epithelial mucosa. Nod2 knockout results in reduced goblet cells in colon mucosa, decreased expression of MUC2 and phospholipase A2, impaired intestinal epithelial barrier function, bacterial translocation and imbalance, and increased susceptibility to colitis and CRC [28,32,33,34,43]. One study showed that gut microbiota richness increases after Nod2 knockout. For example, *Firmicutes*, *Corynebacteriaceae*, *Bdellovibrionaceae,* and *Deferribacteriaceae* were increased [27].	*Firmicutes* *↑* *Proteobacteria* *↑* *Deferribacteres phyla* *↑* *Lachnospiraceae* *↑* *Ruminococcaceae* *↑* *Corynebacteriaceae (Actinobacteria)* *↑* *Bdellovibrionaceae (Proteobacteria)* *↑* *Deferribacteriaceae (Deferribacteres)* *↑*
Nlrp3	Nlrp3 is a cytoplasmic receptor that promotes IL-18 production by non-hematopoietic cells, thereby maintaining gut microbiota homeostasis and the integrity of the intestinal epithelial barrier. After Nlrp3 deletion, the expression of IL-1β, IL-18, IL-10, and TGF-β is decreased, the secretion of antibacterial products is decreased, and the expression of β defensin altered, resulting in the destruction of the intestinal integrity, and altered gut microbiota (*Rikenellaceae*, *Enterobacteriaceae*, *Mycobacterium*, *Clostridium* are increased, *Bacteroidaceae* is decreased) [37,38]. These mice are more likely to develop CRC and have more severe disease manifestations. *Nlrp3*^R258W^ mice have increased Treg cell numbers by increasing IL-1β production and AMPs secretion to reshape gut microbiota composition (such as decreased *Actinobacteria*, *Verrucomicrobia,* and *Akkermansia*, while *Lactobacillus* are increased). Their resistance to colitis and CRC is increased [41].	*Rikenellaceae* ↑ *Enterobacteriaceae* ↑ *Clostridium* ↑ *Lactobacillus* ↑ *Bacteroidaceae* ↓ *Verrucomicrobia* ↓ *Akkermansia* ↓
Nlrp6	Nlrp6 is a cytoplasmic receptor that is highly expressed in intestinal epithelial cells and myeloid-derived immune cells, such as dendritic cells, macrophages, and monocytes [44]. It can regulate mucus secretion of goblet cells in response to bacterial invasion and also regulate microbial colonization by regulating AMPs secretion. After Nlrp6 knockout, secretion of mucus in goblet cells is decreased, secretion of IL-18 is decreased, the ability of intestinal epithelial cells to recover after DSS-induced injury is weakened, and the expression of intestinal downstream AMPs genes is altered, thus increasing the susceptibility to colitis and related tumors. Nlrp6 knockout mice have changes in gut microbiota (*Prevotellaceae*, *TM7*, *Bacteroidetes*, *Proteobacteria* are increased, while *Firmicutes* are decreased); this change could not be repeated in the same littermate control experiment and contradictory results were identified, indicating that unidentified genetic or environmental factors may play a critical role [45,46,47,48,49]. Therefore, the effect of Nlrp6 on the relative abundance of gut microbiota needs to be further verified.	*Prevotellaceae* ↑ *TM7* ↑ *Akkermansia muciniphila* ↑ *Firmicutes* ↓
Nlrp12	Nlrp12 is a cytoplasmic receptor that is primarily expressed in DCs and neutrophils [50]. It can inhibit intestinal inflammation by inhibiting both typical and atypical NF-κB and excessive immune signaling. The activation of NF-κB, ERK, and STAT3 is increased after Nlrp12 deletion, and the diversity and composition of gut microbiota are altered (*Bacteroidales*, *Clostridiales,* and *Lachnospiraceae* are decreased, while *Erysipelotrichaceae* is increased) and dyshomeostasis of the intestinal environment occurs, thereby increasing host susceptibility to colitis and related tumors [51].	*Bacteroides* ↓ *Clostridia* ↓ *Lachnospiraceae* ↓ *Erysipelotrichaceae* ↑
Aim2	Aim2 is a cytosolic innate immune receptor that can inhibit colitis and related CRC by regulating intestinal stem cell proliferation and gut microbiota composition. Specifically, Aim2 mediates IL-18 secretion, which in turn regulates IL-22 secretion and increases the release of AMPs such as Reg3β and Reg3γ to maintain intestinal homeostasis. After Aim2 knockout, intestinal microbial composition is changed significantly (the abundance of *Akkermansia, Muciniphila,* and *Anaeroplasma* is increased, while the abundance of *Anaerostipes*, *Bifidobacterium*, *Flexispira*, *Prevotella,* and *Paraprevotella* is decreased), AKT activation and transcription of genes related to cell proliferation increased, further increasing the host susceptibility to colitis and related tumors [52,53].	*Akkermansia muciniphila* ↑ *Odoribacter* ↑ *Anaeroplasma* ↑ *Anaerostipes* ↓ *Flexispira* ↓ *Paraprevotella* ↓ *Bifidobacterium* ↓ *Prevotella* ↓ *Fusobacterium* ↓
RIG-I	RIG-I is a cytoplasmic RNA sensor. After Rig-I knockout, IgA secretion is decreased, STAT3 phosphorylation is decreased, and Reg3γ secretion is decreased, inducing a gut microbiota disorder, leading to colitis and related tumors. The species richness and diversity of the gut microbiota are increased in *Rig-I−/−* mice, but the bacterial species have not been elucidated. However, antibiotic-treated *Rig-I−/−* mice remain susceptible to colitis, suggesting that gut microbiota disorders are not the primary underlying cause of susceptibility [54]. Therefore, the role of gut microbiota in CRC needs to be further verified.	The species richness and diversity of intestinal microbiota are increased, but the bacterial species have not been characterized.
cGAS/Sting	cGAS/Sting is a cytoplasmic DNA sensor that can be activated by CDNs or cytoplasm dsDNA to recruit TBK1, phosphorylate IRF3 and NF-κB, and induce IFN-Ⅰ production. In *Sting−/−* mouse, intestinal mucosa secretion and IgA production are decreased, cup cells are decreased, the secretion of IL-1β and IL-10 is decreased, and the gut microbiota is altered (*Allobacolum*, *Bifidobacterium,* and *Actinomycetes* are decreased, while *Disulfovibrio* and *Proteus* are increased), increasing the susceptibility to DSS-induced colitis [55,56].	*Disulfovibrio↑* *Proteus↑* *Allobacolum ↓* *Bifidobacterium* ↓ *Actinomycetes* ↓
CLRs	CLRs are localized at the cell surface that recognizes carbohydrates on the surface of fungal pathogens in a Ca^2+^-dependent manner to initiate an antifungal immune response. Loss of CLRs leads to the decreased activation of NF-κB, decreased production of cytokines such as IL-6, TNF-α, and Th17, decreased production of AMPs such as S100A8 and S100A9, decreased repair ability of intestinal epithelial tissue, decreased phagocytosis and bactericidal ability of macrophages, and altered gut microbiota (for example, *Lactobacillus murinus* and *Lactobacillus Johnsonii Gal-2* are increased in the intestinal tract after Dectin-1 deletion, and *Candida Tropicalis* is increased in the intestinal tract after Dectin-3 deficiency.), further aggravating DSS-induced colitis [57,58].	*Dectin-1−/−*: *Lactobacillus murinus* ↑ *Lactobacillus johnsonii GAL-2* ↑ *Dectin-3−/−*: *Candida tropicalis* ↑

Note: ↑ indicates the increased abundance of the bacteria. ↓ indicates the decreased abundance of the bacteria.
